# Supplementary material for: Genome-Wide Analysis of the RbcS Gene Family and Expression Analysis Under Light Response in Brassica napus L
Source: Plants (Basel). 2025 Dec 24;15(1):58. doi: 10.3390/plants15010058 (PMC12787431; doi:10.3390/plants15010058)
Supplement: Supplementary file 1 [file plants-15-00058-s001.zip › Figure. S1.pdf]

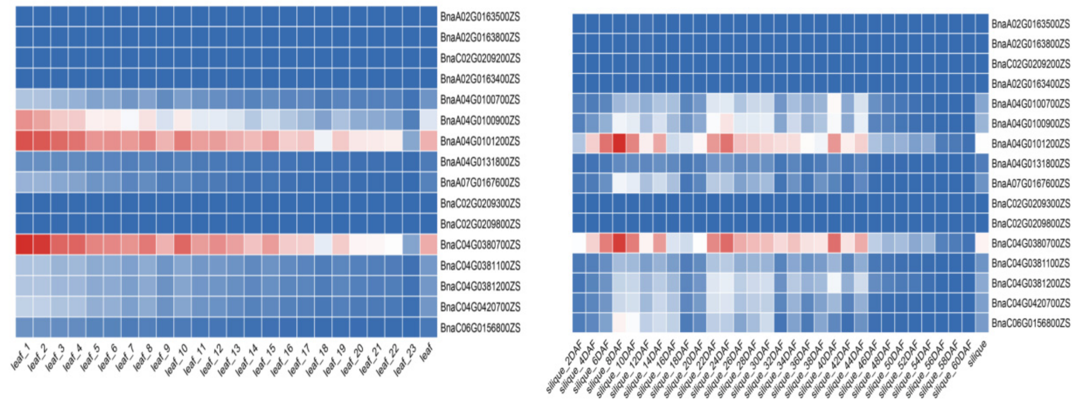

Figure S1. Expression profiles of *BnRbcSs* across leaves and siliques different developmental stages.
