# Supplementary material for: Genome-Wide Analysis of the RbcS Gene Family and Expression Analysis Under Light Response in Brassica napus L
Source: Plants (Basel). 2025 Dec 24;15(1):58. doi: 10.3390/plants15010058 (PMC12787431; doi:10.3390/plants15010058)
Supplement: Supplementary file 1 [file plants-15-00058-s001.zip › Table S1.pdf]

Table S1 The gene abbreviation ID and Sequences of primers used for RT-qPCR

Gene abbreviation ID	Gene name	Primer	Sequence(5'-3')
RbcSA2-1	*BnaA02G0163400ZS*	Forward primer	CATGTAAGGCCAACACCAAAG
		Reverse primer	AGGTCAGGAAGGTAAGAGAGAG
RbcSA2-2	*BnaA02G0163500ZS*	Forward primer	TGTAAGGCGAACACCAAAGT
		Reverse primer	AGGTCAGGAAGGTAAGAGAGAG
RbcSA2-3	*BnaA02G0163800ZS*	Forward primer	GTTATGCTCTCTTCCGCTACAA
		Reverse primer	GGGAAAGCAGCAGATGACTTA
RbcSA2-4	*BnaC02G0209200ZS*	Forward primer	GACCTCTTCTCTGGCTCATTC
		Reverse primer	GTAATTTCGGTGTTGGCCTTAC
RbcSA4-1	*BnaA04G0101200ZS*	Forward primer	CAAGGCCAACAACGACATTAC
		Reverse primer	GGTAAGAGAGGGTCTCAAACTTC
RbcSA4-2	*BnaA04G0100700ZS*	Forward primer	TACCTTCTCCGCAACAAGTG
		Reverse primer	CAGTAACGGCCATCGTAGTATC
RbcSA4-3	*BnaA04G0100900ZS*	Forward primer	TACCTTCTCCGCAACAAGTG
		Reverse primer	CAGTAACGGCCATCGTAGTATC
RbcSA4-4	*BnaA04G0131800ZS*	Forward primer	AAGCAACGGAGGAAGAGTTAG
		Reverse primer	CGTCAGTAAGGTCAGGAAGATAAG
RbcSA7-1	*BnaA07G0167600ZS*	Forward primer	ATCGCAAGCAATGGAGGAA

		Reverse primer	CGTCAGTAAGATCAGGAAGGTAAG
RbcSC2-1	*BnaC02G0209300ZS*	Forward primer	CCGCTACAATGACCTCTTCTC
		Reverse primer	GTAATTTCGGTGTTGGCCTTAC
RbcSC2-2	*BnaC02G0209800ZS*	Forward primer	TTAAGTCCTCTGCTGCTTTCC
		Reverse primer	CATCGGTAAGGTCAGGAAGATAAG
RbcSC4-1	*BnaC04G0380700ZS*	Forward primer	TACCTTCTCCGCAACAAGTG
		Reverse primer	CAGTAACGGCCATCGTAGTATC
RbcSC4-2	*BnaC04G0381100ZS*	Forward primer	CAAGCAACGGAGGAAGAGTTAG
		Reverse primer	CGGTAAGGTCAGGAAGGTAAGA
RbcSC4-3	*BnaC04G0381200ZS*	Forward primer	CTAGCAACGGAGGAAGAGTTAG
		Reverse primer	CAACTTCGGTAAGGTCAGGAA
RbcSC4-4	*BnaC04G0420700ZS*	Forward primer	TGCAAACAACGGAGGAAGA
		Reverse primer	CGTCAGTAAGGTCAGGAAGATAAG
RbcSC6-1	*BnaC06G0156800ZS*	Forward primer	TGGCAAGCAATGGAGGAA
		Reverse primer	CGTCAGTAAGATCAGGAAGGTAAG
	ACTIN	Forward primer	TGTGCTTGACTCTGGTGATGGT
		Reverse primer	GACGGAGGATAGCGTGAGGAAG
